# A key genomic signature associated with lymphovascular invasion in head and neck squamous cell carcinoma

**DOI:** 10.1186/s12885-020-06728-1

**Published:** 2020-03-30

**Authors:** Jian Zhang, Huaming Lin, Huali Jiang, Hualong Jiang, Tao Xie, Baiyao Wang, Xiaoting Huang, Jie Lin, Anan Xu, Rong Li, Jiexia Zhang, Yawei Yuan

**Affiliations:** 1 Department of Radiation Oncology, Affiliated Cancer Hospital & Institute of Guangzhou Medical University, State Key Laboratory of Respiratory Diseases, Guangzhou Institute of Respiratory Disease, Guangzhou, 510095 P. R. China; 2 The First Tumor Department, Maoming People’s Hospital, Maoming, 525000 P. R. China; 3 Department of Cardiovascularology, Tungwah Hospital of Sun Yat-sen University, Dongguan, 523000 P. R. China; 4 Department of Urology, Tungwah Hospital of Sun Yat-sen University, Dongguan, 523000 P. R. China; 5 State Key Laboratory of Respiratory Disease, National Clinical Research Center for Respiratory Disease, Guangzhou Institute of Respiratory Health, the First Affiliated Hospital of Guangzhou Medical University, Guangzhou, 510120 P. R. China

**Keywords:** Lymphovascular invasion, Head and neck squamous cell carcinoma, Hub genes, TCGA, Weighted gene co-expression network analysis

## Abstract

**Background:**

Lymphovascular invasion (LOI), a key pathological feature of head and neck squamous cell carcinoma (HNSCC), is predictive of poor survival; however, the associated clinical characteristics and underlying molecular mechanisms remain largely unknown.

**Methods:**

We performed weighted gene co-expression network analysis to construct gene co-expression networks and investigate the relationship between key modules and the LOI clinical phenotype. Functional enrichment and KEGG pathway analyses were performed with differentially expressed genes. A protein–protein interaction network was constructed using Cytoscape, and module analysis was performed using MCODE. Prognostic value, expression analysis, and survival analysis were conducted using hub genes; GEPIA and the Human Protein Atlas database were used to determine the mRNA and protein expression levels of hub genes, respectively. Multivariable Cox regression analysis was used to establish a prognostic risk formula and the areas under the receiver operating characteristic curve (AUCs) were used to evaluate prediction efficiency. Finally, potential small molecular agents that could target LOI were identified with DrugBank.

**Results:**

Ten co-expression modules in two key modules (turquoise and pink) associated with LOI were identified. Functional enrichment and KEGG pathway analysis revealed that turquoise and pink modules played significant roles in HNSCC progression. Seven hub genes (CNFN, KIF18B, KIF23, PRC1, CCNA2, DEPDC1, and TTK) in the two modules were identified and validated by survival and expression analyses, and the following prognostic risk formula was established: [risk score = EXP_DEPDC1_ * 0.32636 + EXP_CNFN_ * (− 0.07544)]. The low-risk group showed better overall survival than the high-risk group (*P* < 0.0001), and the AUCs for 1-, 3-, and 5-year overall survival were 0.582, 0.634, and 0.636, respectively. Eight small molecular agents, namely XL844, AT7519, AT9283, alvocidib, nelarabine, benzamidine, L-glutamine, and zinc, were identified as novel candidates for controlling LOI in HNSCC (*P* < 0.05).

**Conclusions:**

The two-mRNA signature (CNFN and DEPDC1) could serve as an independent biomarker to predict LOI risk and provide new insights into the mechanisms underlying LOI in HNSCC. In addition, the small molecular agents appear promising for LOI treatment.

## Background

Head and neck squamous cell carcinoma (HNSCC) is one of the most common cancers with high morbidity and mortality rates worldwide; > 90% of head and neck cancers are squamous cell carcinomas that arise in the oral cavity, oropharynx, and larynx [1]. Metastasis is the main cause of treatment failure and an important factor affecting prognosis [2]. Thus, elucidating the underlying genomic changes seems valuable for controlling lymph node metastases.

In case of HNSCC, advanced TNM stage, histological grade, and lymph node status, which are well-known major risk factors of metastatic disease and poor overall survival (OS) and disease-free survival, are poor prognostic indicators [3–5]. Lymphovascular invasion (LOI) has been associated with lymph node metastasis in HNSCC [6–8]. Thus, identification of effective molecular prognosticators of LOI should be a useful way to decrease the risk of metastasis in patients with HNSCC.

According to recent studies, the clinical characteristics of and parameters contributing to LOI remain uncertain. In fact, the incidence of LOI in patients with HNSCC is highly inconsistent, varying from 14 to 47% [9, 10]. This considerable variation can be attributed to small sample sizes, distribution differences, and heterogeneity of HNSCC. Therefore, it is imperative to conduct clinical studies with large sample sizes to analyze the genomic and clinical characteristics of LOI. This should, consequently, facilitate the development of novel therapeutic targets, enhancing the survival of HNSCC patients with LOI.

The Cancer Genome Atlas (TCGA) has generated comprehensive, multidimensional maps of key genomic changes in several types of cancers, including HNSCC, and provided histopathological annotations and clinical survival data relevant to patients with HNSCC over a follow-up duration of 10 years. This has enabled the systematic evaluation of the relationship between LOI and gene signatures, providing clarity on key gene modules involved in LOI in patients with HNSCC. This has in turn provided us with comprehensive, systemic understanding of LOI not only at the genomic but also at the prognostic level.

## Methods

### Patient selection and data pre-processing

Data pertaining to patients with HNSCC were downloaded from TCGA database. RNA expression profiles and clinical survival data of 500 patients were obtained (Table 1). Among them, clinical prognosis data of 339 patients were available. According to the threshold of |logFC| > 1 and *P* < 0.05, 2248 genes that met the criteria were identified as differentially expressed genes (DEGs) (Additional file 1: Table S1). The intersection of DEGs based on the NCBI Gene (Additional file 2: Table S2) and Online Mendelian Inheritance in Man (OMIM) (Additional file 3: Table S3) databases was performed using the Venn Diagram package in the R language.

**Table 1:** Clinicopathological characteristics of 500 patients with HNSCC

Parameters	Subtype	Patients
Age (years)	> 61	234
	≤61	265
	Unknow	1
Gender	Male	367
	Female	133
Lymphovascular invasion	Yes	120
	No	219
	Unknow	161
Pathologic stage	Stage I-II	125
	Stage III-IV	337
	Unknow	68
OS duration (months)	< 1	8
	≥1	491
	Unknow	1

### Construction of the co-expression network

Based on mRNA expression data, the scale-free gene modules of co-expression were constructed using weighted gene co-expression network analysis (WGCNA) [11, 12]. To ensure reliability of the co-expression network, hierarchical clustering was performed based on Euclidean distance, and two outlier samples were removed. Module–trait associations were considered to be important clinical characteristics between the clinical phenotype and module eigengenes (MEs). We analyzed the module–trait correlation and determined relevant modules, which were closely associated with the LOI clinical phenotype. An adequate soft-threshold power that met the scale-free topology criterion was selected for transforming the former correlation matrix into an adjacency matrix, which was subsequently converted into a Topological Overlap Matrix (TOM) using the “TOM similarity” function in R. TOM-based dissimilarity was computed as measure distance, and an mRNA clustering dendrogram and module colors were obtained. In the clustering dendrogram, the minimum module size and cut height were separately set to 30 and 0.25, respectively. For key gene modules, gene significance and module membership indicated a positive correlation level between RNA expression profiles and the LOI clinical phenotype and between RNA expression profiles and clinical MEs.

### Enrichment analysis of key co-expression modules

As per previously reported methodology [12, 13], aberrantly expressed mRNAs in key gene modules were selected, and gene ontology (GO) function and Kyoto Encyclopedia of Genes and Genomes (KEGG) pathway analyses were performed. For the former, corresponding genes were classified into the biological process (BP) category, and for the latter, genes within key co-expression modules were used to detect the function of gene modules. *P* < 0.05 indicated statistical significance.

### Protein–protein interaction (PPI) network analysis and hub gene identification

As previously reported [14, 15], key gene co-expression modules were further explored to predict gene function correlation using the STRING database (confidence score > 0.9). Cytoscape was employed to screen significant gene pairs in the PPI network [16]. We further screened the PPI network modules by performing Molecular Complex Detection (MCODE) analysis. The criteria of MCODE were as follows: degree cutoff = 2, node cutoff = 0.2, maximum depth = 100, and k-score = 2. Finally, 24 genes were selected as hub genes and further analyzed using univariate survival analysis. Seven genes with significant prognostic differences were selected as characteristic genes, with *P* < 0.05.

### Survival analysis of hub genes

According to the expression profiles of characteristic genes, Kaplan–Meier analysis was performed to explore prognostic differences; Cox proportional hazard ratio and 95% confidence interval were used for analysis. *P* < 0.05 indicated statistical significance. The least absolute shrinkage and selection operator (LASSO) model was then used to identify vital mRNAs from the prognostic hub genes. The LASSO method was utilized by the package “glmnet” in the R (version 3.5.1) software.

### mRNA expression analysis

We used GEPIA (http://gepia.cancer-pku.cn/), a web-based tool that delivers fast and customizable functionalities based on TCGA and Genotype–Tissue Expression data, to analyze mRNA expression levels of the seven hub genes [17].

### Immunohistochemistry analysis

To validate the protein expression levels of the seven hub genes, as per the method reported by Jian et al. [18], we used the Human Protein Atlas database (https://www.proteinatlas.org/) (HNSCC samples = 519, normal tissue samples = 44; scale bar = 200 μm). All captured images were manually annotated by certified pathologists.

### Establishment of prognostic risk score formula

In light of the expression level of the hub genes and regression coefficients, a prognostic risk formula was established by multivariable Cox regression analysis. A risk score was calculated for each patient using this formula. All patients were consequently classified into a high- and low-risk group by utilizing the median risk score as the cutoff value. Next, the Kaplan–Meier survival curve was used to compare prognosis between the low- and high-risk groups. Moreover, a time-dependent receiver operating characteristic (ROC) curve was applied to assess diagnostic accuracy based on the risk score for 1-, 3-, and 5-year OS probability. *P* < 0.05 indicated statistical significance.

### Identification of small molecular drugs

DrugBank is a comprehensive, high-quality, freely accessible, online database that combines quantitative drug data and target information [19]. The turquoise and pink modules in the PPI network were mapped onto the DrugBank database. |Connectivity score| > 2 was used as the cutoff value to identify small molecular drugs that could target HNSCC.

### Statistical analysis

Univariate analysis was performed using SPSS 17.0 (SPSS Inc., Chicago, IL, USA). Cumulative survival time was calculated and analyzed by the Kaplan–Meier and log-rank test. Differences between the groups were tested by the chi-square or Fisher’s exact test. *P* < 0.05 was considered statistically significant.

## Results

### WGCNA and key module analysis

The initial quality was assessed using the average linkage method. Two outlier samples were removed after the clustering. The remaining 339 HNSCC and 44 normal tissue samples with clinical information pertaining to LOI were used for subsequent analyses. In total, 2601 genes showed the highest variance via the average linkage/hierarchical clustering method.

**Fig. 1:**
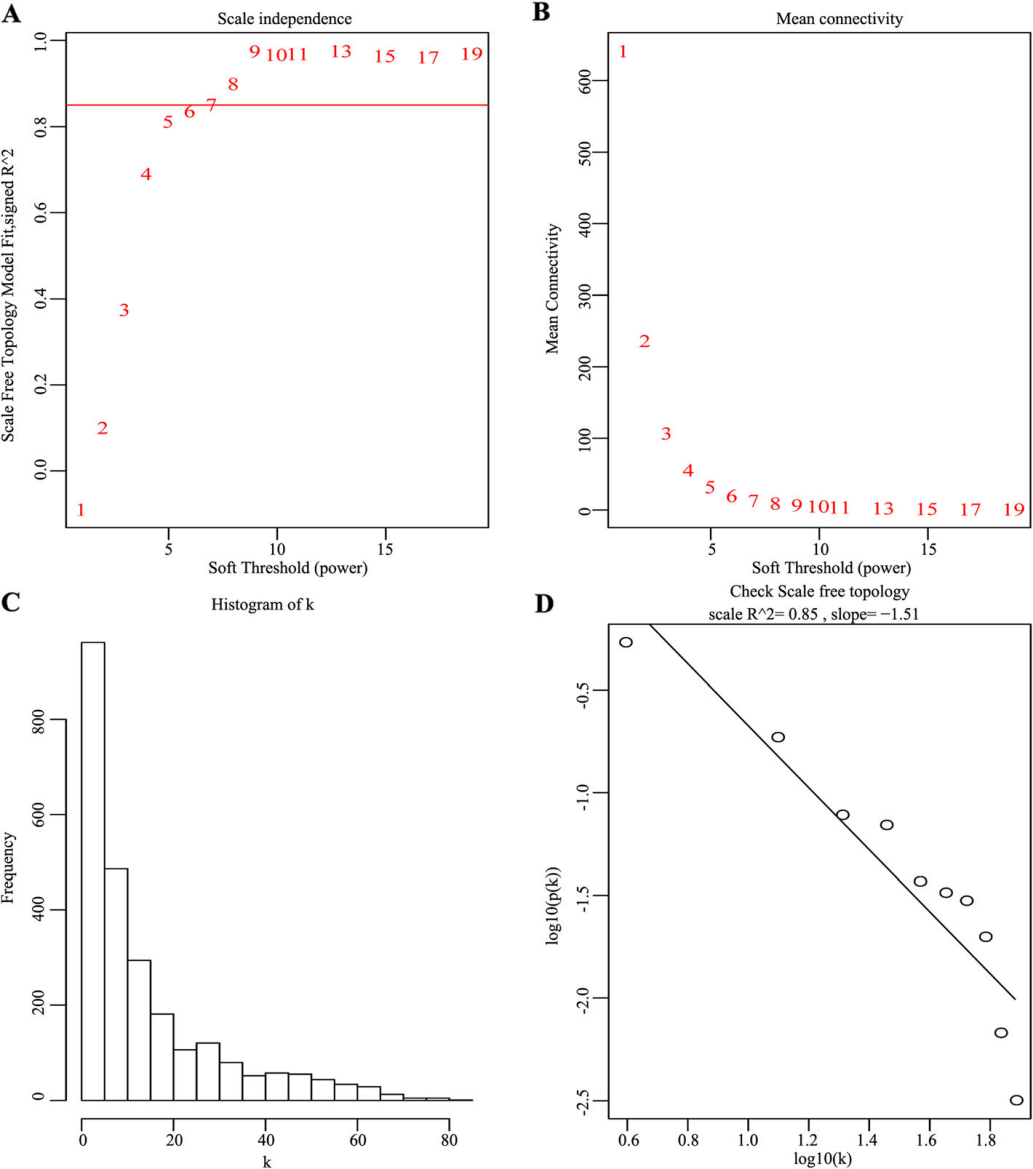
Determination of soft-threshold power in WGCNA. **a** Scale-free index analysis for soft-threshold power (β) in HNSCC. **b** Mean connectivity analysis for various soft-threshold powers. **c** Histogram depicting connectivity distribution when β = 7. **d** Checking scale-free topology when β = 7

**Fig. 2:**
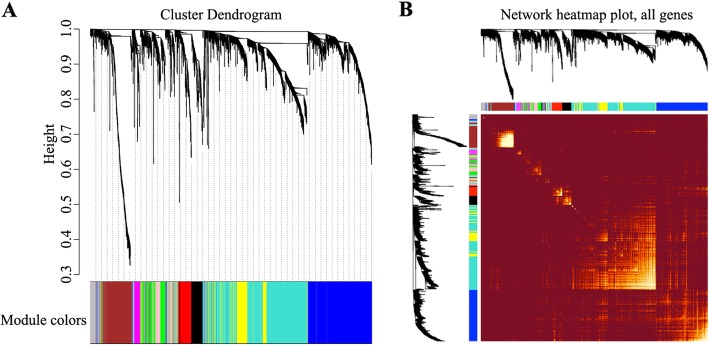
Visualization of WGCNA results. **a** mRNA clustering dendrogram obtained by hierarchical clustering of Topological Overlap Matrix (TOM)-based dissimilarity, with the corresponding module colors indicated by colored rows. Each colored row represents a color-coded module containing a group of highly connected mRNAs. Each color represents a module in the constructed gene co-expression network. **b** The heatmap depicts TOM among all genes in WGCNA. Light color represents low overlap and progressively darker color represents higher overlap

**Fig. 3:**
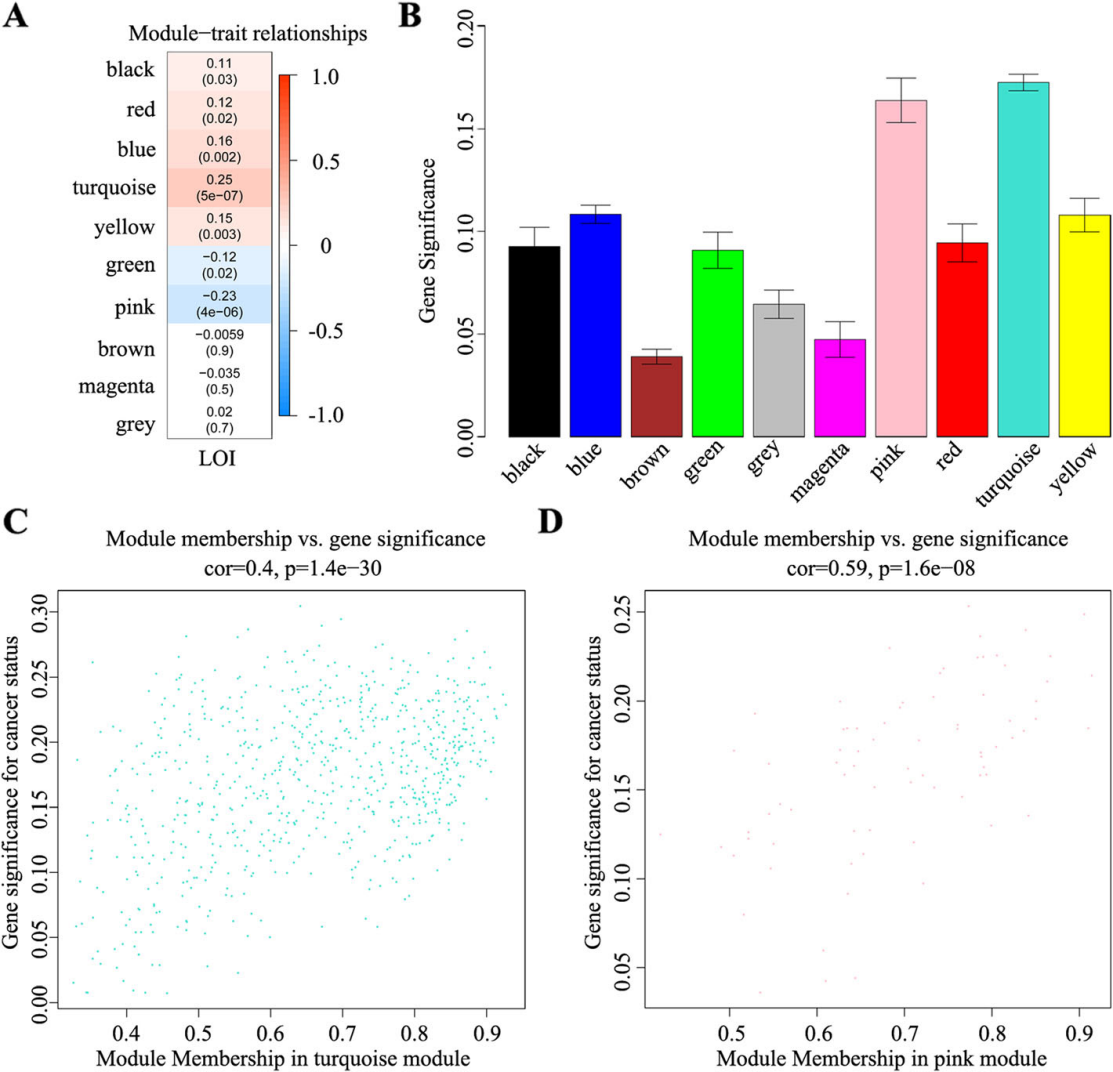
Correlation analysis of module–trait associations and clinical characteristics. **a** The column corresponds to the LOI phenotypic trait. Heatmap of each cell contains the *P* value of that module and the LOI phenotypic trait. Correlations between the turquoise module with the LOI phenotypic trait (cor = 0.25; *P* = 5E− 07) and the pink module with the LOI phenotypic trait (cor = − 0.23; *P* = 4E− 06) were significant. **b** Bar plot of the significance level of 10 co-expression modules associated with LOI status. **c** and **d** Correlation analysis between gene significance of LOI status and module membership in the turquoise **(c)** and pink **(d)** modules

To establish a scale-free network, the scale-free index (Fig. 1a) and mean connectivity (Fig. 1b, c) were calculated. We found that when the power value of β = 7, the scale-free topology for the fitting index reached 0.85 (Fig. 1d). Different genes were subsequently grouped into modules according to the association of expression. Moreover, genes with similar expression patterns were placed into different modules via average linkage clustering. Finally, a total of 10 modules were identified (Fig. 2). On exploring the correlation between the MEs and LOI clinical phenotype, we found that 10 co-expression modules were correlated with the LOI clinical phenotype (Fig. 3a) and were associated with cancer status, particularly turquoise and pink key modules (Fig. 3b). Then, scatter diagrams were constructed for correlation analyses between gene significance for LOI status and module membership in the turquoise (Fig. 3c) and pink (Fig. 3d) modules, which revealed that genes in the two modules were significantly related with LOI status. The correlation and *P* values (Fig. 3c, d) indicated that the turquoise and pink modules showed high correlations with LOI status.

### Enrichment analysis of key co-expression modules

**Fig. 4:**
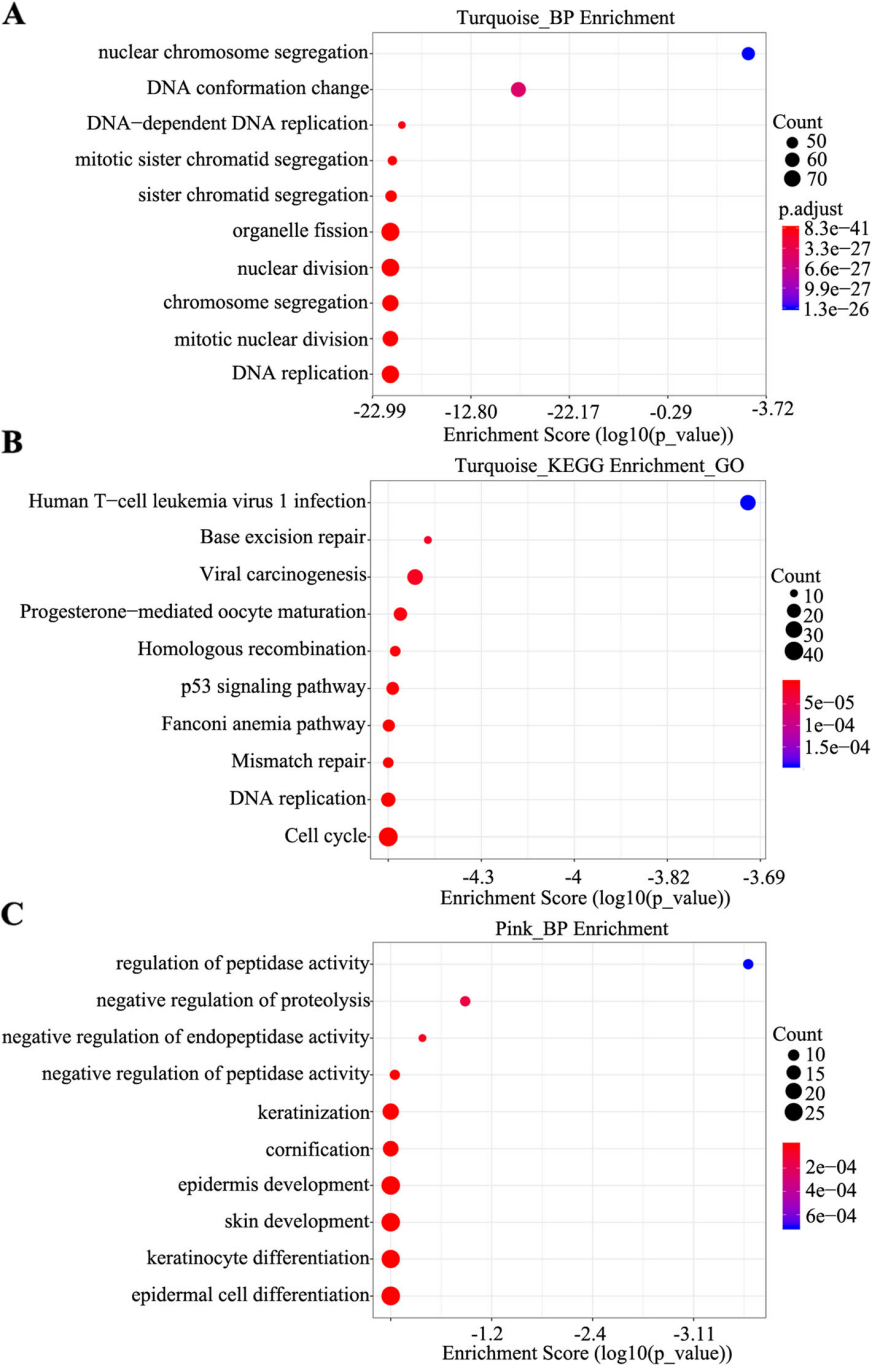
GO function and KEGG pathway analyses. **a** GO enrichment analysis of the turquoise module in the biological process category. **b** GO enrichment analysis of the turquoise module in the KEGG pathway. **c** GO enrichment analysis of the pink module in the biological process category

To determine the function of genes in the key co-expression modules, GO function and KEGG pathway analyses were performed. GO function analysis showed that the turquoise module was associated with DNA replication, mitotic nuclear division, chromosome segregation, nuclear division, and DNA-dependent DNA replication, whereas KEGG pathway analysis indicated that it was associated with cell cycle, DNA replication, mismatch repair, and p53 signaling pathway (*P* < 0.05) (Fig. 4a, b). Similarly, GO function analysis indicated that the pink module was involved in not only squamous cell functions, such as epidermal cell differentiation, keratinocyte differentiation, skin development, epidermis development, and cornification (*P* < 0.05), but also regulation of protein secretion, for example, via the negative regulation of proteolysis, peptidase activity, and endopeptidase activity (*P* < 0.05) (Fig. 4c). These results indicated that the turquoise and pink modules played a pivotal role in LOI in patients with HNSCC.

### PPI network analysis and hub genes

**Fig. 5:**
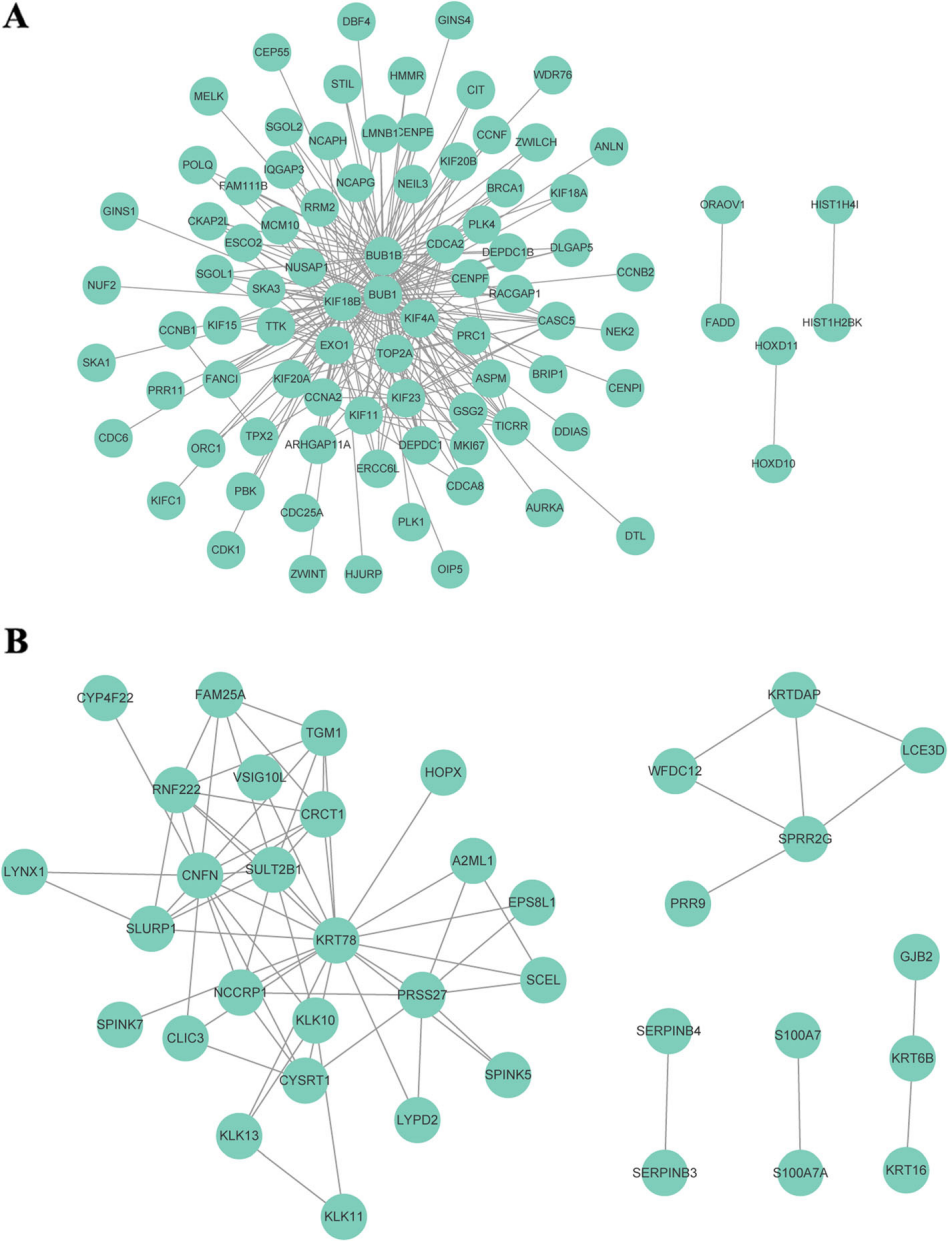
Hub genes identified by the PPI network. **a** and **b** PPI network interaction of DEGs in the turquoise **(a)** and pink **(b)** modules

To identify hub genes in the key modules, PPI network analysis was performed using the STRING database. Connection threshold was used to define the hub genes; 89 genes, including the top five genes KIF18B, BUB1, BUB1B, KIF4A, and EXO1, in the turquoise module (connect threshold > 0.25) and 38 genes, including the top five genes KRT78, CNFN, SLURP1, PRSS27, and CRCT1, in the pink module (connect threshold > 0.10) were screened as candidate hub genes (Fig. 5, Additional file 4: Table S4, Additional file 5: Table S5). In addition, connect degree (> 6) was used to define the hub genes, which led to the identification of 24 hub genes (18 in the turquoise module and 6 in the pink module).

### Prognostic value and expression analysis of hub genes

**Fig. 6:**
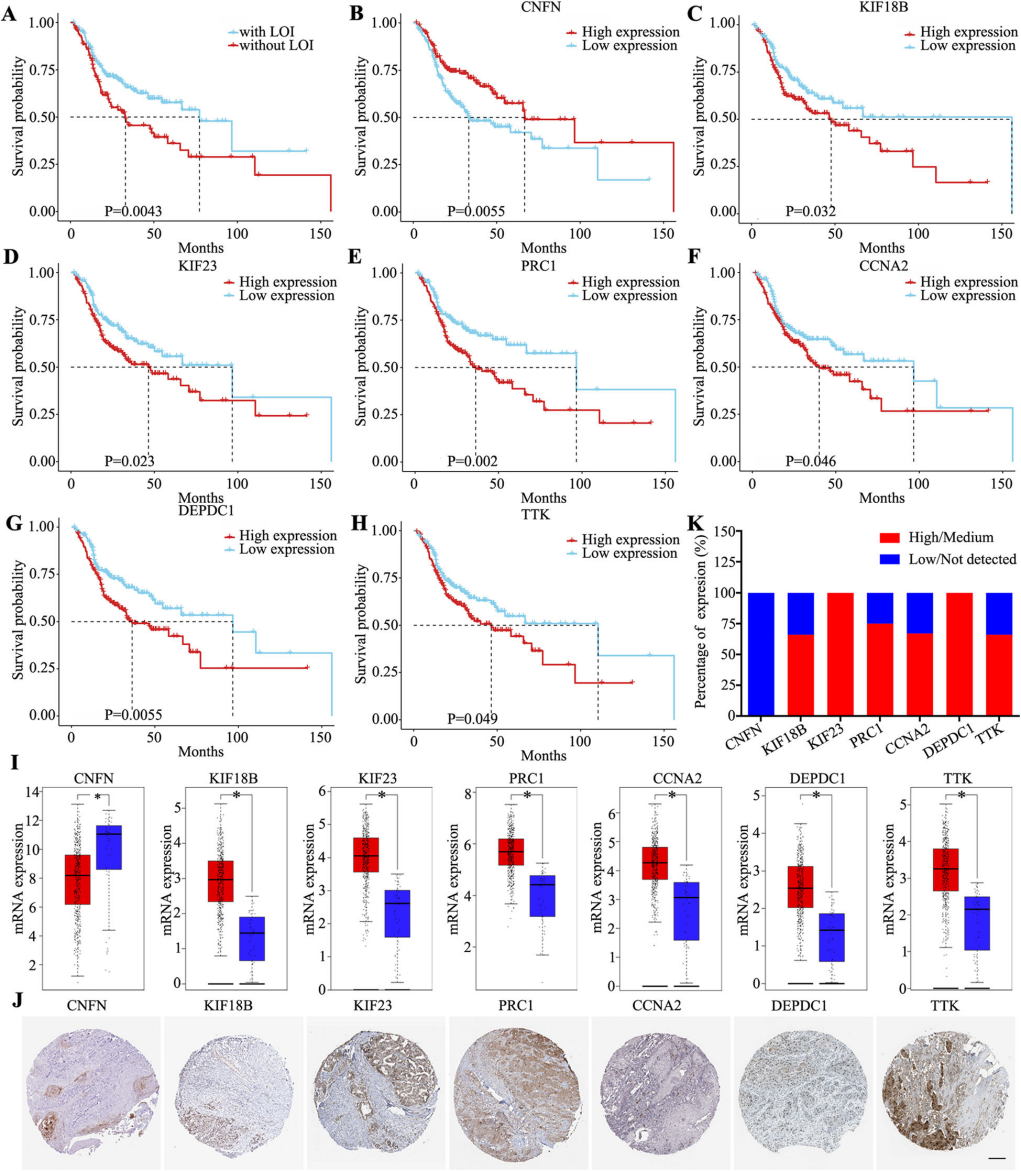
Prognostic value and expression analysis of seven hub genes in HNSCC. **a** Ten-year cumulative survival of HNSCC patients with or without LOI. **b–h** Ten-year survival analysis of CNFN (**b**), KIF18B (**c**), KIF23 (**d**), PRC1 (**e**), CCNA2 (**f**), DEPDC1 (**g**), and TTK (**h**). **i** mRNA expression levels of the seven hub genes in HNSCC samples (*n* = 519, red box) and normal tissue samples (*n* = 44, blue box) based on GEPIA. **j** Immunohistochemistry images of the seven hub genes based on the Human Protein Atlas database. **k** Protein expression levels analyzed by immunohistochemistry based on the Human Pathology Atlas database. **P < 0.01 and *P < 0.05

After excluding samples with no survival information or survival duration < 1 month, 339 HNSCC samples were used to evaluate the prognosis of the 24 hub genes. We found that HNSCC samples with LOI showed a poor clinical outcome than those without LOI (*P* < 0.05), indicating that LOI is a key histological characteristic in HNSCC (Fig. 6a). Univariate survival analysis was then performed using the R-package survival, and the results indicated that CNFN was associated good survival (Fig. 6a) but KIF18B, KIF23, PRC1, CCNA2, DEPDC1, and TTK were associated with poor survival in HNSCC samples with LOI (*P* < 0.05; Fig. 6c–h).

To determine mRNA expression levels of the seven hub genes (CNFN, KIF18B, KIF23, PRC1, CCNA2, DEPDC1, and TTK), we used GEPIA and found that CNFN was significantly downregulated but KIF18B, KIF23, PRC1, CCNA2, DEPDC1, and TTK were significantly upregulated in HNSCC (*P* < 0.05; Fig. 6i). To assess protein expression levels of the seven hub genes, we performed protein expression analyses using the HPA database (Fig. 6j). The expression level of CNFN was low and thus could not be detected (100%, *n* = 4), whereas that of KIF18B (66.7%, *n* = 3), KIF23 (100%, n = 4), PRC1 (75.0%, n = 4), CCNA2 (66.7%, n = 3), DEPDC1 (100%, n = 3), and TTK (66.7%, n = 3) was either moderate or high (Fig. 6k).

### Establishment of the prognostic risk score formula

**Fig. 7:**
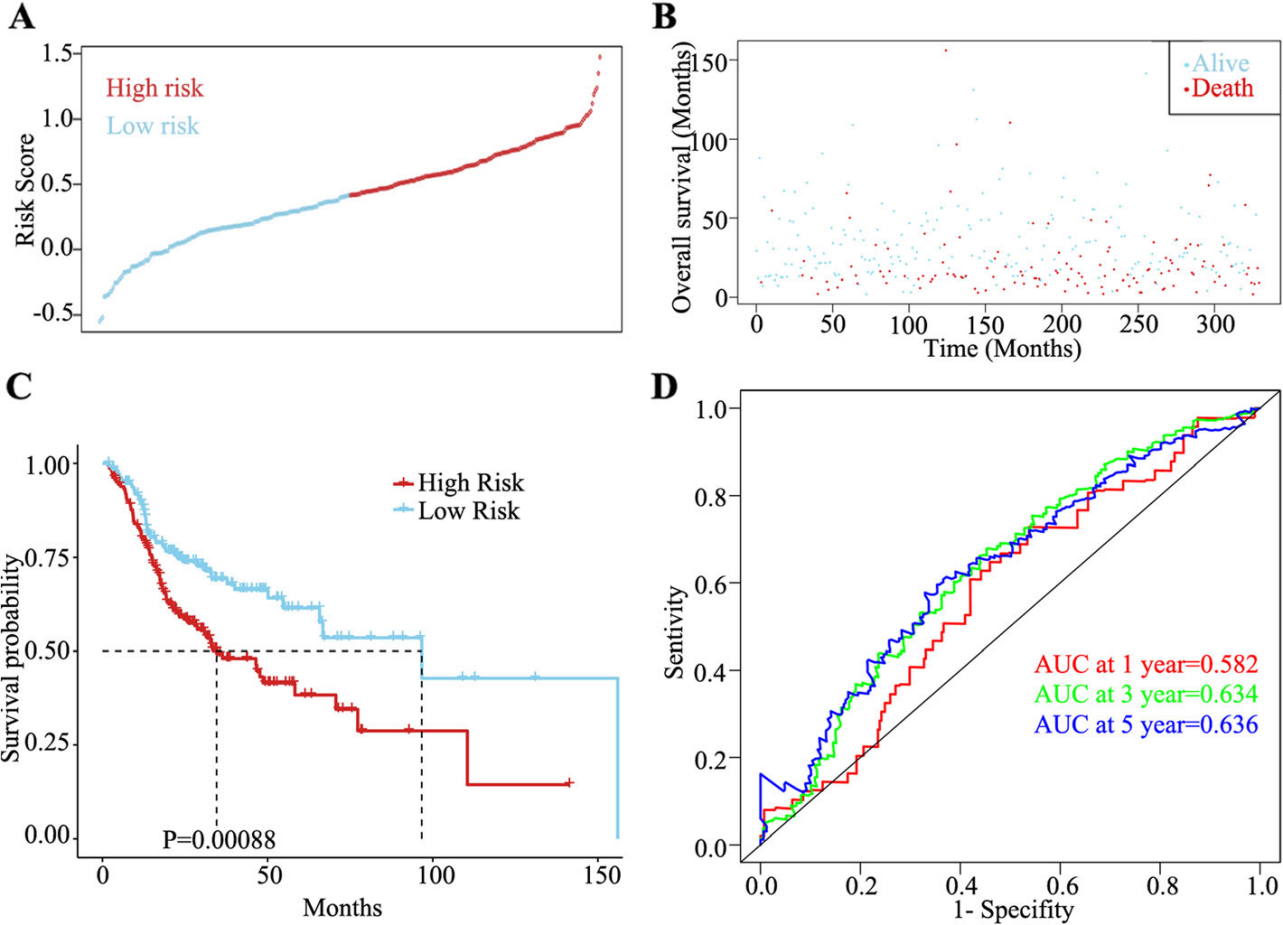
Distribution of risk score, survival status, and time-dependent ROC analysis of the integrated two-mRNA signature. **a** Risk score distribution **b** Overall survival (OS) status of 330 patients. **c** Kaplan–Meier curve of OS between the low- and high-risk groups split by the median risk score. **d** Time-dependent ROC analysis for 1-, 3-, and 5-year OS probability

Using the LASSO method and multivariable Cox regression analysis, two mRNAs (CNFN and DEPDC1) were identified as integrated prognostic biomarkers in patients with HNSCC. We then established a prognostic risk score formula based on the expression profiles of CNFN and DEPDC1 and their regression coefficients. The prognostic risk score formula was as follows: risk score = EXP_DEPDC1_ * 0.32636 + EXP_CNFN_ * (− 0.07544). The risk score was calculated for all patients, classifying patients into the high-risk (*n* = 165) and low-risk (*n* = 165) group using the median risk score as the cutoff value (Additional file 6: Table S6). The distribution of risk scores and survival status of patients are shown in Fig. 7a and b, respectively.

We then assessed the prognostic value of the aforementioned formula using Kaplan–Meier analysis. Patients in the low-risk group showed better OS than those in the high-risk group (*P* < 0.001; Fig. 7c). Moreover, time-dependent ROC analysis was utilized to evaluate the prognostic capacity of the formula. The areas under the ROC curve for 1-, 3-, and 5-year OS were 0.582, 0.634, and 0.636, respectively, implying that the integrated two-mRNA signature was much better at predicting the risk of LOI in patients with HNSCC (Fig. 7d).

### Identification of small molecular agents

To determine which small molecular agents in the turquoise and pink modules could target LOI, we searched all drug–gene interactions in the DrugBank database. |Connectivity score| > 2 and *P* < 0.05 were used for screening; we found that five drug–module interactions (XL844, AT7519, AT9283, alvocidib, and nelarabine) in the turquoise module and three drug–module interactions (benzamidine, L-glutamine, and zinc) in the pink module could be used to target LOI (*P* < 0.05; Table 2). To investigate the clinical application of the eight small molecular agents in head and neck cancer or solid tumor, we examined the clinical trial registration of these agents using ClinicalTrials.gov (https://clinicaltrials.gov/ct2/home). Although a study on benzamidine remains to be conducted, three clinical trials of L-glutamine (NCT03015077, NCT02282839, NCT00006994) and zinc (NCT00036881, NCT03531190, NCT02868151) in head and neck cancer have been conducted (Additional file 7: Table S7). Moreover, XL844 (NCT00475917), AT7519 (NCT00390117, NCT02503709), AT9283 (NCT00443976, NCT00985868), alvocidib (NCT00080990), and nelarabine (NCT01376115) have been explored in the context of solid tumor/cancer. These findings indicated that XL844, AT7519, AT9283, alvocidib, nelarabine, benzamidine, L-glutamine, and zinc appear promising for treating LOI.

**Table 2:** Significantly enriched small molecular agents

Module	Drug	Connection	***P***
Pink	Benzamidine	2	8.29E−07
Pink	L-Glutamine	2	1.28E−05
Pink	Zinc	2	0.001325331
Turquoise	XL844	2	0
Turquoise	AT7519	2	0
Turquoise	AT9283	2	0
Turquoise	Alvocidib	5	3.55E−06
Turquoise	Nelarabine	2	0.000198791

## Discussion

Metastasis is the leading cause of treatment failure in patients with HNSCC [20]. Nodal metastatic disease is an independent factor for poor survival in HNSCC [21–23]. Several clinicopathological parameters have been associated with nodal metastasis, such as tumor size [9], tumor depth [24], tumor differentiation [25], histological grade [26], and LOI [4]. Herein we performed comprehensive, integrative genomic analyses of LOI in patients with HNSCC from the molecular to clinical and prognostic levels. We established a novel two-mRNA signature for predicting LOI risk in HNSCC. The survival curves indicated that the low- and high-risk groups stratified by the mRNA signature had a significant difference in prognoses. Time-dependent ROC analysis revealed that the mRNA signature had a high accuracy in predicting OS. Moreover, the small molecular agents, namely XL844, AT7519, AT9283, alvocidib, nelarabine, benzamidine, L-glutamine, and zinc, were identified as novel candidates for treating LOI.

With the application of sequencing techniques, genomic studies have transitioned from assessing aberrant expression levels of individual genes to systematically integrating omics data from cancer tissues. The molecular mechanisms underlying LOI remain unclear. TCGA database has been used by several studies to define the genomic landscape of HNSCC, providing us an opportunity to integrate genomics data and understand molecular changes associated with LOI. In the current study, we constructed a co-expression network module of HNSCC and found that the turquoise and pink modules were significantly associated with LOI. Functional enrichment analysis indicated that the key gene modules were involved in not only squamous cell functions, such as epidermal cell differentiation, keratinocyte differentiation, skin development, epidermis development, and cornification but also regulation of protein secretion, for instance, via the negative regulation of proteolysis, peptidase activity, and endopeptidase activity. Furthermore, the turquoise module was associated with DNA replication, mitotic nuclear division, nuclear division, and DNA-dependent DNA replication. KEGG pathway analysis validated that the turquoise module was associated with cell cycle, DNA replication, mismatch repair, and p53 signaling pathway, indicating the involvement of pertinent genes in LOI in patients with HNSCC.

Lymphatic vessels are remodeled by the tumor microenvironment, including cancer cells, mutations of oncogenic driver genes, and interactions between immune checkpoint signals and their receptors [27]. Herein we systematically analyzed the mRNA expression level of 339 HNSCC samples with LOI and 44 normal tissue samples; 2522 DEGs were identified. PPI network and module analyses showed that 18 genes in the turquoise module (e.g., KIF18B, BUB1, BUB1B, KIF4A, and EXO1) and six genes in the pink module (e.g., KRT78, CNFN, SLURP1, PRSS27, and CRCT1) were associated with LOI in HNSCC. However, the roles and mechanisms of these 24 genes in the metabolic and immune reprogramming of the tumor microenvironment demand further exploration.

Early diagnosis of LOI is pivotal, considering that timely treatment is of utmost importance in HNSCC patients with LOI [28, 29]. Despite the development and application of magnetic resonance imaging and positron emission tomography–computed tomography to assess LOI in HNSCC, the detection rate of early-stage LOI remains low [30]. In this study, the hub genes in the key modules related to LOI were screened, and prognostic value and expression analyses showed that CNFN was downregulated and associated with good prognosis, whereas KIF18B, KIF23, PRC1, CCNA2, DEPDC1, and TTK were upregulated and associated with poor prognosis. The two-mRNA signature could stratify the risk of LOI and predict OS in patients with HNSCC; however, there are also some limitations. First, the two-mRNA signature needs to be further explored. Second, the prognostic value of the mRNA panel was not very satisfactory and thus demands additional investigation. Finally, the biological functions and mechanisms of the two mRNAs were not assessed in this study.

Although the targeted treatment for LOI is lacking and unreliable, DrugBank provides comprehensive molecular information pertaining to drugs and their targets for treating LOI. Based on interactions between the drugs and key modules, we found eight small molecular agents (benzamidine, L-glutamine, zinc, XL844, AT7519, AT9283, alvocidib, and nelarabine) that could target LOI. AT7519 and alvocidib, a cyclin-dependent kinase inhibitor, have been reported to target CDK1 and thus proposed to have anticancer effects [31–35]. XL844 is a specific inhibitor of checkpoint kinase-1 and -2 and prevents the formation of a normal mitotic spindle; it can reportedly effectively sensitize cancer cells to induce cell cycle arrest [36]. Clinical trial registration analyses of the eight small molecular agents indicated that they have been widely explored in head and neck cancer or solid tumor. These results indicated that they could be used for treating LOI in patients with HNSCC; however, their roles and mechanisms in the context of LOI require further exploration.

## Conclusions

To summarize, we herein performed a comprehensive, integrative genomic analysis of LOI in patients with HNSCC and established a two-mRNA signature that could stratify the risk of LOI and predict OS. Finally, we report that benzamidine, L-glutamine, zinc, XL844, AT7519, AT9283, alvocidib, and nelarabine are novel candidate drugs for controlling LOI in HNSCC.

## Supplementary information

**Additional file 1: Table S1.** Differentially expressed genes in TCGA database.

**Additional file 2: Table S2.** HNSCC-related genes in the NCBI Gene database.

**Additional file 3: Table S3.** HNSCC-related genes in the OMIM database.

**Additional file 4: Table S4.** Candidate hub genes in the turquoise module.

**Additional file 5: Table S5.** Candidate hub genes in the pink module.

**Additional file 6: Table S6.** Risk score.

**Additional file 7: Table S7.** Clinical trials of small molecular agents.

## Data Availability

All data were downloaded from TCGA (https://cancergenome.nih.gov/), OMIM (https://www.omim.org/), NCBI Gene (https://www.ncbi.nlm.nih.gov/gene/), and DrugBank (https://www.drugbank.ca/) databases.

## Abbreviations

LOI: Lymphovascular invasion
HNSCC: Head and neck squamous cell carcinoma
DEGs: Differentially expressed genes
MEs: Module eigengenes
WGCNA: Weighted gene co-expression network analysis
MCODE: Molecular complex detection

## References

[1] Torre LA, Bray F, Siegel RL, Ferlay J, Lortet-Tieulent J, Jemal A (2015). Global cancer statistics, 2012. CA Cancer J Clin.

[2] Warnakulasuriya S (2009). Global epidemiology of oral and oropharyngeal cancer. Oral Oncol.

[3] Vasan K, Low TH, Gupta R, Ashford B, Asher R, Gao K, Ch'ng S, Palme CE, Clark JR (2018). Lymph node ratio as a prognostic factor in metastatic cutaneous head and neck squamous cell carcinoma. Head Neck..

[4] Wreesmann VB, Katabi N, Palmer FL, Montero PH, Migliacci JC, Gönen M, Carlson D, Ganly I, Shah JP, Ghossein R (2016). Influence of extracapsular nodal spread extent on prognosis of oral squamous cell carcinoma. Head Neck..

[5] Liu SA, Wang CC, Jiang RS, Lee FY, Lin WJ, Lin JC (2017). Pathological features and their prognostic impacts on oral cavity cancer patients among different subsites - a singe institute's experience in Taiwan. Sci Rep.

[6] Moore BA, Weber RS, Prieto V, El-Naggar A, Holsinger FC, Zhou X, Lee JJ, Lippman S, Clayman GL (2005). Lymph node metastases from cutaneous squamous cell carcinoma of the head and neck. Laryngoscope..

[7] Yilmaz T, Hosal AS, Gedikoglu G, Onerci M, Gürsel B (1998). Prognostic significance of vascular and perineural invasion in cancer of the larynx. Am J Otolaryngol.

[8] Karahatay S, Thomas K, Koybasi S, Senkal CE, Elojeimy S, Liu X, Bielawski J, Day TA, Gillespie MB, Sinha D (2007). Clinical relevance of ceramide metabolism in the pathogenesis of human head and neck squamous cell carcinoma (HNSCC): attenuation of C (18)-ceramide in HNSCC tumors correlates with lymphovascular invasion and nodal metastasis. Cancer Lett.

[9] Kurokawa H, Yamashita Y, Takeda S, Zhang M, Fukuyama H, Takahashi T (2002). Risk factors for late cervical lymph node metastases in patients with stage I or II carcinoma of the tongue. Head Neck..

[10] Hahn SS, Spaulding CA, Kim JA, Constable WC (1987). The prognostic significance of lymph node involvement in pyriform sinus and supraglottic cancers. Int J Radiat Oncol Biol Phys.

[11] Lu JM, Chen YC, Ao ZX, Shen J, Zeng CP, Lin X, Peng LP, Zhou R, Wang XF, Peng C (2019). System network analysis of genomics and transcriptomics data identified type 1 diabetes-associated pathway and genes. Genes Immu.

[12] Yuan L, Chen L, Qian K, Qian G, Wu CL, Wang X, Xiao Y (2017). Co-expression network analysis identified six hub genes in association with progression and prognosis in human clear cell renal cell carcinoma (ccRCC). Genom Data.

[13] Zhang Y, Wang J, Ji LJ, Li L, Wei M, Zhen S, Wen CC (2017). Identification of key gene modules of neuropathic pain by co-expression analysis. J Cell Biochem.

[14] von Mering C, Huynen M, Jaeggi D, Schmidt S, Bork P, Snel B (2003). STRING: a database of predicted functional associations between proteins. Nucleic Acids Res.

[15] Xia WX, Yu Q, Li GH, Liu YW, Xiao FH, Yang LQ, Rahman ZU, Wang HT, Kong QP (2019). Identification of four hub genes associated with adrenocortical carcinoma progression by WGCNA. Peer J.

[16] Shannon P, Markiel A, Ozier O, Baliga NS, Wang JT, Ramage D, Amin N, Schwikowski B, Ideker T (2003). Cytoscape: a software environment for integrated models of biomolecular interaction networks. Genome Res.

[17] Tang Z, Li C, Kang B, Gao G, Li C, Zhang Z (2017). GEPIA: a web server for cancer and normal gene expression profiling and interactive analyses. Nucleic Acids Res.

[18] Zhang J, Zheng Z, Zheng J, Xie T, Tian Y, Li R, Wang B, Lin J, Xu A, Huang X (2019). Epigenetic-mediated Downregulation of zinc finger protein 671 (ZNF671) predicts poor prognosis in multiple solid tumors. Front Oncol.

[19] Wishart DS, Feunang YD, Guo AC, Lo EJ, Marcu A, Grant JR, Sajed T, Johnson D, Li C, Sayeeda Z, Assempour N (2018). DrugBank 5.0: a major update to the DrugBank database for 2018. Nucleic Acids Res.

[20] Leeman JE, Li JG, Pei X, Venigalla P, Zumsteg ZS, Katsoulakis E, Lupovitch E, McBride SM, Tsai CJ, Boyle JO (2017). Patterns of treatment failure and Postrecurrence outcomes among patients with locally advanced head and neck squamous cell carcinoma after Chemoradiotherapy using modern radiation techniques. JAMA Oncol.

[21] Layland MK, Sessions DG, Lenox J (2005). The influence of lymph node metastasis in the treatment of squamous cell carcinoma of the oral cavity, oropharynx, larynx, and hypopharynx: N0 versus N+. Laryngoscope..

[22] Sessions DG, Spector GJ, Lenox J, Parriott S, Haughey B, Chao C, Marks J, Perez C (2000). Analysis of treatment results for floor-of-mouth cancer. Laryngoscope..

[23] Sessions DG, Lenox J, Spector GJ, Chao C, Chaudry OA (2003). Analysis of treatment results for base of tongue cancer. Laryngoscope..

[24] Pentenero M, Gandolfo S, Carrozzo M (2005). Importance of tumor thickness and depth of invasion in nodal involvement and prognosis of oral squamous cell carcinoma: a review of the literature. Head Neck.

[25] Byers RM, El-Naggar AK, Lee YY, Rao B, Fornage B, Terry NH, Sample D, Hankins P, Smith TL, Wolf PJ (1998). Can we detect or predict the presence of occult nodal metastases in patients with squamous carcinoma of the oral tongue?. Head Neck..

[26] Umeda M, Yokoo S, Take Y, Omori A, Nakanishi K, Shimada K (1992). Lymph node metastasis in squamous cell carcinoma of the oral cavity: correlation between histologic features and the prevalence of metastasis. Head Neck..

[27] Achen MG, Stacker SA (2008). Molecular control of lymphatic metastasis. Ann N Y Acad Sci.

[28] Solares CA, Mason E, Panizza BJ (2016). Surgical Management of Perineural Spread of head and neck cancers. J Neurol Surg B Skull base.

[29] Bur AM, Lin A, Weinstein GS (2016). Adjuvant radiotherapy for early head and neck squamous cell carcinoma with perineural invasion: a systematic review. Head Neck..

[30] Lee H, Lazor JW, Assadsangabi R, Shah J (2019). An Imager's guide to Perineural tumor spread in head and neck cancers: radiologic footprints on (18) F-FDG PET, with CT and MRI correlates. J Nucl Med.

[31] Dolman ME, Poon E, Ebus ME, den Hartog IJ, van Noesel CJ, Jamin Y, Hallsworth A, Robinson SP, Petrie K, Sparidans RW (2015). Cyclin-dependent kinase inhibitor AT7519 as a potential drug for MYCN-dependent neuroblastoma. Clin Cancer Res.

[32] Kang MA, Kim W, Jo HR, Shin YJ, Kim MH, Jeong JH (2018). Anticancer and radiosensitizing effects of the cyclin-dependent kinase inhibitors, AT7519 and SNS032, on cervical cancer. Int J Oncol.

[33] Kong Y, Sheng X, Wu X, Yan J, Ma M, Yu J, Si L, Chi Z, Cui C, Dai J (2017). Frequent genetic aberrations in the CDK4 pathway in Acral melanoma indicate the potential for CDK4/6 inhibitors in targeted therapy. Clin Cancer Res.

[34] Hafner M, Mills CE, Subramanian K, Chen C, Chung M, Boswell SA, Everley RA, Liu C, Walmsley CS, Juric D (2019). Multiomics profiling establishes the Polypharmacology of FDA-approved CDK4/6 inhibitors and the potential for differential clinical activity. Cell Chem Biol.

[35] Roskoski R (2019). Cyclin-dependent protein serine/threonine kinase inhibitors as anticancer drugs. Pharmacol Res.

[36] Matthews DJ, Yakes FM, Chen J, Tadano M, Bornheim L, Clary DO, Tai A, Wagner JM, Miller N, Kim YD (2007). Pharmacological abrogation of S-phase checkpoint enhances the anti-tumor activity of gemcitabine in vivo. Cell Cycle.

